# Hybrid Resonators and Highly Tunable Terahertz Metamaterials Enabled by Vanadium Dioxide (VO_2_)

**DOI:** 10.1038/s41598-017-04692-8

**Published:** 2017-06-28

**Authors:** Shengxiang Wang, Lei Kang, Douglas H. Werner

**Affiliations:** 1 School of Electronic and Electrical Engineering, Wuhan Textile University, Wuhan, Hubei 430073 People’s Republic of China; 2 Department of Electrical Engineering, The Pennsylvania State University, University Park, PA 16802 USA

## Abstract

Hybrid metamaterials that exhibit reconfigurable responses under external stimulus, such as electric fields and light radiation, have only recently been demonstrated by combining active media with patterned metallic structures. Nevertheless, hybrid terahertz (THz) metamaterials whose spectral performance can be dynamically tuned over a large scale remain rare. Compared with most active media (for instance, silicon) that provide limited activity, vanadium dioxide (VO_2_), which exhibits an insulator-to-metal transition, has been recently explored to facilitate dynamically tunable metamaterials. More importantly, the phase transition yields a three orders of magnitude increase in THz electrical conductivity, which suggests the potential for creating VO_2_ based hybrid resonators that operate at THz frequencies. Here, we show that an integration of VO_2_ structures and conventional metallic resonating components can enable a class of highly tunable THz metamaterials. Considering the widely studied phase-transition dynamics in VO_2_, the proposed hybrid metamaterials are capable of offering ultrafast modulation of THz radiation.

## Introduction

Metamaterials represent a new class of engineered materials that are generally composed of an array of subwavelength ‘artificial atoms’ that provide an unprecedented ability for electromagnetic wave manipulation. Recent developments have been focused on functionalizing metamaterials by endowed them with tunability at the ‘atomic’ level^1–3^. Various active media (*e*.*g*., semiconductors^4–10^, liquid crystals^11–18^, ferromagnetic^19^ and ferroelectric^20^ materials and graphene^21–24^) that are capable of providing variable dielectric or magnetic properties have been introduced into metamaterial systems to facilitate a reconfigurable response. In particular, tunable terahertz (THz) metamaterials^6–10, 17, 18, 25–29^ have received considerable attention due to their ability to provide highly efficient manipulation of THz radiation. Nevertheless, effective tuning of hybrid THz metamaterials has been considerably constrained by the limited range in the active material properties that can be achieved in practice. For example, the response modulation observed in refs 6, 7 was attributed to the electrically triggered carrier density change of 10^16^ cm^−3^ in n-type GaAs and the optical pumping induced conductivity varying up to 10^2^ Ω^−1^ cm^−1^ in silicon, respectively. Furthermore, despite the radically different regulating mechanism, only mild responses to an applied electric field were identified in graphene^28^ and nematic liquid crystal^17^ integrated tunable THz metamaterials.

Vanadium dioxide (VO_2_), a classical transition metal oxide that undergoes an insulator-to-metal transition (IMT) near room temperature (*T*_IMT_ ~ 67 °C), has been intensively studied with respect to the fundamental physics involved during the transition process, *e*.*g*., the femtosecond structural dynamics^30^, and the potential applications, *e*.*g*., next generation transistors^31^. As an active medium that has been demonstrated to be sensitive to thermal, optical and electric fields, VO_2_ was recently utilized to enable active metamaterials at both THz^32–38^ and optical frequencies^39–43^. For instance, taking advantage of the hysteresis characteristic of VO_2_, Driscoll and coworkers have demonstrated electrically controlled frequency tuning as well as the memory effect of a VO_2_ integrated THz metamaterial^32^. From a different standpoint, Liu and coworkers have reported the observation of a THz-field-induced IMT in VO_2_ metamaterials^37^, in which the split ring resonators (SRRs) offer both the locally-enhanced nonlinearity and the globally macroscopic observation of the IMT dynamics. A close examination of the reported THz properties of VO_2_ reveals that IMT leads to an approximately three orders of magnitude increase in the conductivity (σ_1_) and, more importantly, when VO_2_ is in its fully metallic state σ_1_ reaches ~4 × 10^3^ Ω^−1^ cm^−1^, which is just one order of magnitude lower than that of gold. In stark contrast to the methodology previously adopted to introduce active media for the achievement of tunability, the sufficiently high conductivity of VO_2_ actually enables the material to support strong THz resonances that can be exploited to achieve highly tunable THz devices. Recently, a temperature controlled THz metamaterial consisting of VO_2_ cut-wires was reported with large transmission amplitude modulation^44^.

In this study, by integrating structured VO_2_ with conventional metallic resonating components, we propose and demonstrate a series of hybrid highly tunable terahertz metamaterials. Furthermore, the work reported here provides a path forward to the design of VO_2_ based THz metadevices. Besides the dramatic manipulation of the response, we also envision that the unique characteristics of the phase transition of VO_2_, including the hysteresis and ultrafast dynamics, may provide a paradigm that can be extended to sophisticated control of THz radiation.

## Results

Despite the elusive microscopic mechanism behind the phase transition, VO_2_ indeed provides a huge contrast in material properties between its insulator and metallic phases. For instance, Kim and coworkers have reported that the exceedingly large conductivity change during IMT of VO_2_ can be utilized to build high-speed next-generation transistors^31^. It should be noted that in this type of electronic application, the electrical current filaments that arise from a localized transition are responsible for the observed resistance magnitude jumps in the voltage-current curves. Optically, VO_2_ during transition should be treated as a composite material with permittivity that can be described by effective medium approximations. Indeed, using scanning near-field infrared microscopy, Qazilbash and coworkers have illustrated the percolation progress at the nanometer scale in VO_2_ thin films, *i*.*e*., the portion of metallic state experiences gradual growth with increasing temperature until a complete transition is achieved^45^. Consequently, the interactions between VO_2_ films and THz waves are in general dominated by their macroscopic material properties. Various groups have reported the temperature-dependent THz conductivity (σ_1_) of VO_2_ thin films deposited on a sapphire substrate^37, 46^. An approximately three orders of magnitude increase in σ_1_ indicates the potential of VO_2_ as an active medium for tunable metamaterials, while, more importantly, the high conductivity exhibited in the metallic state indicates the possibility of further creating hybrid VO_2_/metal THz resonators and metamaterials with a high degree of tunability.

**Figure 1:**
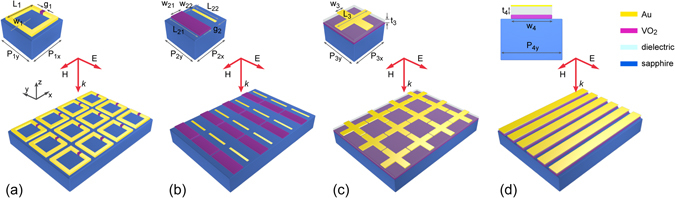
Vanadium dioxide enables hybrid THz metamaterials. Schematic showing four different metamaterial examples, each consisting of an array of hybrid resonators. These metamaterial designs include (**a**) gap-loaded split ring resonators (SRRs), (**b**) asymmetric VO_2_/Au double-bars, (**c**) Au-cross/VO_2_ absorber structures, and (**d**) Au/VO_2_ paired-strips. The geometrical parameters (all unit in μm) are: *P*_1x_ = *P*_1y_ = 30, *L*_1_ = 25, *w*_1_ = 3 and *g*_1_ = 1.5; *P*_2x_ = 75, *P*_2y_ = 72, *L*_21_ = 70, *L*_22_ = 55, *w*_21_ = 25, *w*_22_ = 5, and *g*_2_ = 20; *P*_3x_ = *P*_3y_ = 100, *L*_3_ = 95, *w*_3_ = 20 and *t*_3_ = 8; *P*_4y_ = 90, *w*_4_ = 60 and *t*_4_ = 12. The thickness of VO_2_ in (**a**) is 200 nm and that in (**b**), (**d**) and (**d**) is 3 μm. The thickness of gold in all designs is 200 nm.

In this paper, we propose a hybridization of VO_2_ microstructures with conventional metallic resonating components that will enable highly-active THz metamaterials. Schematics of the four types of VO_2_ integrated hybrid metamaterials that will be considered here are illustrated in Fig. 1. In particular, the IMT property of VO_2_ is expected to enable THz radiation manipulation through various mechanisms. Here we consider four different examples, which include tuning the resonance of gap-loaded SRRs (Fig. 1(a)), producing Fano resonances in asymmetric VO_2_/Au double-bars (Fig. 1(b)), creating a perfect absorber effect in Au-cross/VO_2_ sandwich structures (Fig. 1(c)), and Au/VO_2_ coupled strips capable of supporting a magnetic resonance (Fig. 1(d)). The corresponding unit cell is depicted as an inset at the left-top corner of each figure. It should be noted that, although sputtering based growth of VO_2_ on different substrates has been reported, crystalline sapphire wafers are preferred to obtain high quality VO_2_ thin films because of the beneficial lattice matching effect^47^. Therefore, without loss of generality, sapphire is assumed as the substrate with a permittivity of 10.5 for all simulations in this work. By fitting the measured THz spectroscopy results reported in ref. 37, we obtain the material properties of VO_2_ as a function of σ_1_ and utilize them to explore the tuning behavior in the proposed hybrid resonators during the IMT process (details available in Methods).

**Figure 2:**
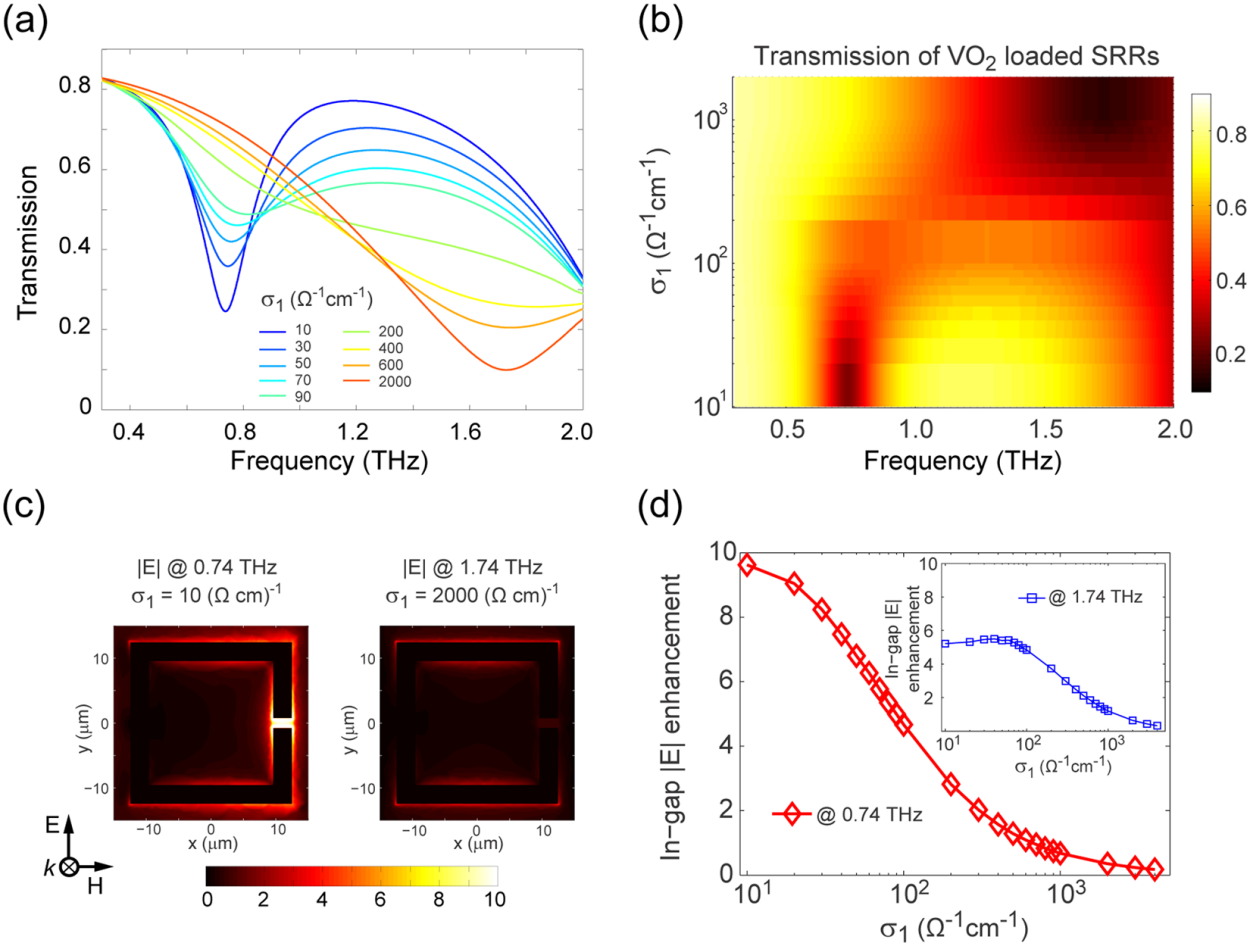
Active performance of the THz metamaterial consisting of an array of SRRs with VO_2_ loaded in their gaps. (**a**) Simulated transmission spectra for a series of VO_2_ conductivity (σ_1_) values. (**b**) A semi-log two-dimensional plot of the transmission spectra. (**c**) The electric field distribution for the first resonance at 0.74 THz when σ_1_ = 10 Ω^−1^ cm^−1^ and the second resonance at 1.74 THz when σ_1_ = 2000 Ω^−1^ cm^−1^. (**d**) The in-gap electric field enhancement as a function of σ_1_ at 0.74 THz. The field enhancement at 1.74 THz is shown in the inset. All data for |E| was normalized to the electric field magnitude of the incident wave.

It has been widely reported that the resonance associated with SRRs, the most-studied and intriguing meta-atom structure, can be efficiently regulated by varying the dielectric environment in the vicinity of their gaps owing to the highly localized field enhancement. Consequently, we first consider an array of SRRs with VO_2_ islands loaded in their gaps, as depicted in Fig. 1(a). The transmission spectra corresponding to a THz plane wave incident on the structure for a series of VO_2_ conductivity values are illustrated in Fig. 2(a). In the case where σ_1_ = 10 Ω^−1^ cm^−1^, which corresponds to VO_2_ at room temperature^37^, a transmission dip indicating a sharp resonance is identified around 0.8 THz. It is evident that the transmission around this resonance increases dramatically with a corresponding increase in σ_1_, while the gradual emergence of a transmission dip around 1.7 THz indicates the onset of a new resonance mode. This is clearly revealed by the electric field distributions (Fig. 2(c)) at the resonances frequencies, *i*.*e*., 0.74 and 1.74 THz. To better display the transmission tuning behavior due to the change in the localized material properties in the gap, a semi-log plot of the σ_1_-dependent THz transmission spectra in the frequency range of interest is shown in Fig. 2(b), which unambiguously indicates a drastic but continuous spectrum tuning before saturation. Furthermore, by monitoring the local field, we study the field enhancement (|E|) in the SRR gap and display the results as a function of σ_1_ in Fig. 2(d). The significant decrease of the on-resonance in-gap field enhancement in the semi-log plots further discloses the tuning effect arising from the phase transition of the critically-placed VO_2_. In other words, the tunability exhibited in Fig. 2 is based upon the change in the electrical properties of the SRRs. Beyond that, as we discussed above, the highly conductive metallic-phase VO_2_ can actually support strong THz resonance. As depicted in Fig. 1(b–d), hereinafter we present three types of hybrid resonators for versatile THz metamaterials that exhibit remarkable response modulation enabled by the phase transition of VO_2_.

Optical Fano resonances, which typically give rise to an asymmetric line-shape of the scattering spectrum, have recently been intensively studied due to their potential applications in optoelectronic devices such as sensors, lasers and switches. In general, the interference between different parts of a Fano system is responsible for the asymmetric resonance profile, leading to the unique enhanced light-matter interactions. Owing to their unprecedented flexibility in manipulating electromagnetic waves, metamaterials are ‘predisposed’ to possessing Fano resonances^48^. Various meta-atoms, such as asymmetric SRRs^49^, SRR-bar structures^50^, dolmen structures^51^, gold heptamers^52^, and so on, have been utilized to realize Fano resonances from microwave to optical frequencies. Among those with different structural complexity, the asymmetric double-bar design^53, 54^, based upon the interference between dipole and quadrupole modes, is more attractive due to its rather simple structure that can support Fano resonances at optical frequencies. By taking advantage of the Fano resonance enabled enhanced nonlinear effect, Moritake and coauthors have demonstrated the controllable fluorescence emission of a quantum dot (QD) embedded in a gold asymmetric double-bar metamaterial^55^.

**Figure 3:**
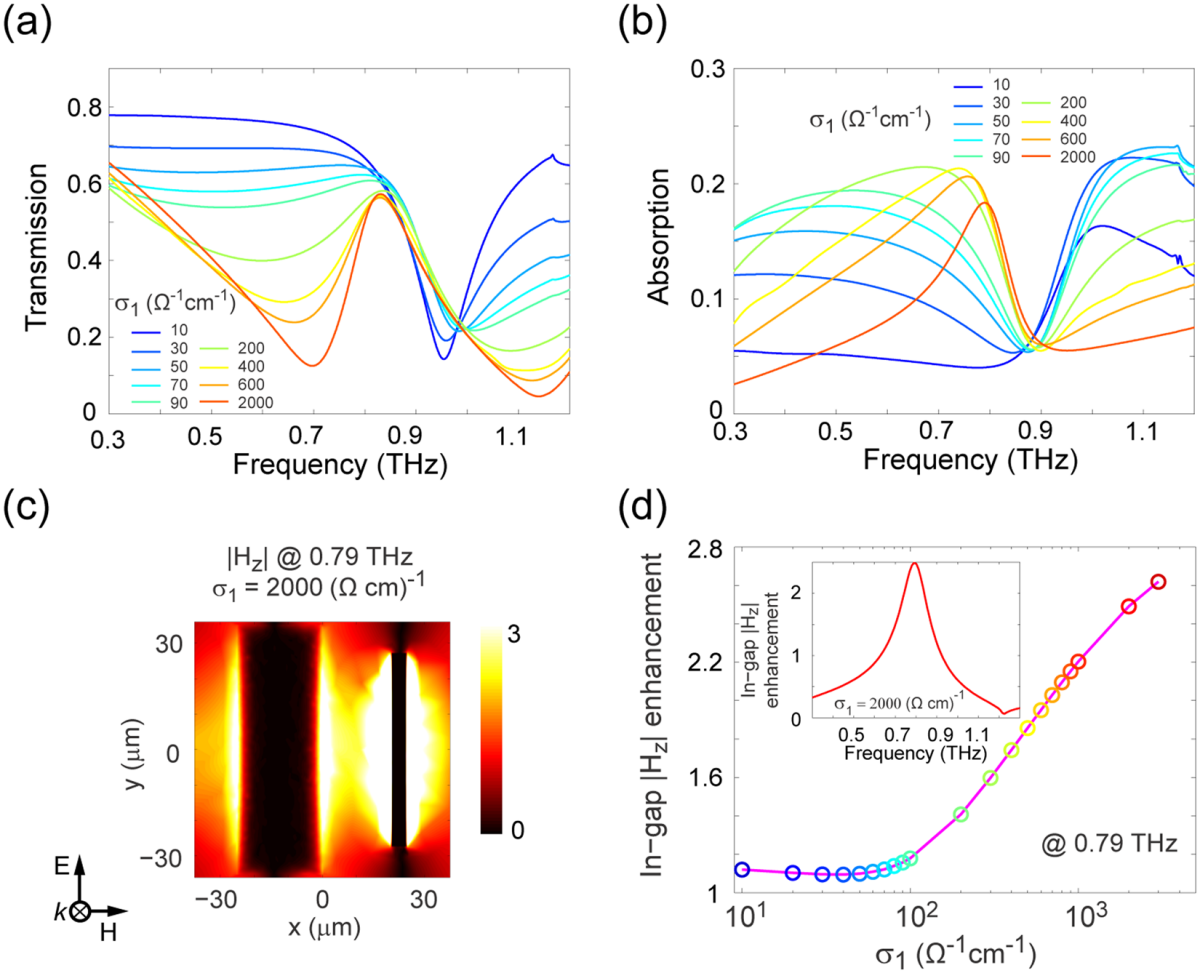
Asymmetric VO_2_/Au double-bar resonator based hybrid metamaterial for a tunable THz Fano resonance. Simulated (**a**) transmission and (**b**) absorption spectra of the hybrid metamaterial for a series of conductivity values σ_1_. (**c**) Magnetic field distribution at 0.79 THz when σ_1_ = 2000 Ω^−1^ cm^−1^. (**d**) The in-gap |H_z_| enhancement as a function of σ_1_ at 0.79 THz. The case for σ_1_ = 2000 Ω^−1^ cm^−1^ as a function of frequency is shown in the inset. All data for |H_z_| was normalized to the magnetic field magnitude of the incident wave.

Fano systems that can be tuned in a dynamic fashion have been reported with, however, limited tuning capability^50, 56^. As the schematic in Fig. 1(b) illustrates, we propose that a metamaterial consisting of an array of asymmetric VO_2_/Au double-bar resonators is a good candidate for demonstrating active tuning of THz Fano resonances. Figure 3(a) and (b) show simulated transmission and absorption spectra, respectively, of the hybrid metamaterial at a series of conductivity values σ_1_ for VO_2_. It can be seen that by increasing the value of σ_1_, a Fano resonance with asymmetric line-shape is gradually identified within the frequency range of interest. Specifically, with VO_2_ in its insulator phase, the dipole resonance of the gold bar produces a sharp transmission dip around 0.95 THz, while, when VO_2_ is highly conductive, the absorption peak appearing around 0.8 THz clearly reveals the characteristic asymmetric profile of a Fano resonance. It can easily be concluded that the VO_2_ bar in its metallic phase supports a dipolar resonance (not shown), whereas the observed Fano resonance arises from the interference between the asymmetric VO_2_/gold double bars. This Fano resonance mode is expected to form non-radiative magnetic resonance and, accordingly, leads to an enhanced magnetic field in the gap between the bars. At σ_1_ = 2000 Ω^−1^ cm^−1^, this is readily identified from the magnetic field distribution at the frequency of the absorption peak (0.79 THz) shown in Fig. 3(c). To further confirm the observed Fano resonance mode, the dependence of the in-gap |H_z_| enhancement as a function of σ_1_ is shown in Fig. 3(d), while the dispersion of the in-gap |H_z_| is depicted in the inset. The results indicate that a Fano resonance starts to emerge when σ_1_ is greater than 100 Ω^−1^ cm^−1^ and, with VO_2_ in its metallic phase, the hybrid system can support a relatively high quality factor Fano resonance. By exhibiting the strong and continuously controllable Fano-like interference, the proposed hybrid asymmetric system promises the potential to achieve controllable slow-light, sensing and other nonlinear THz devices.

**Figure 4:**
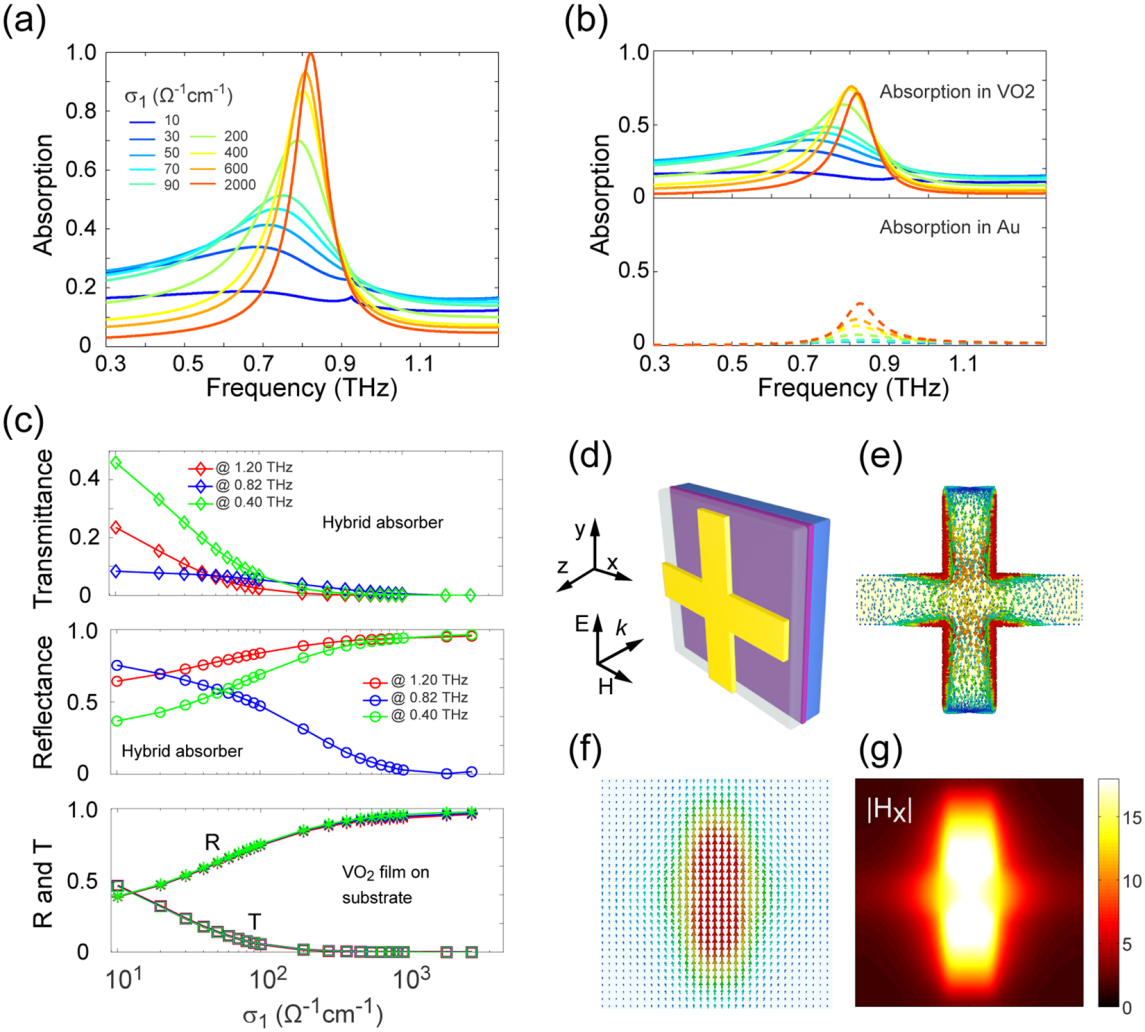
VO_2_ based tunable THz metamaterial absorber. Simulated (**a**) absorption spectra of the hybrid metamaterial and (**b**) the absorption in the VO_2_ and gold material. (**c**) Transmittance (*T*) and reflectance (*R*) of the hybrid metamaterial and a bare 3-μm-thick VO_2_ film on a substrate at the resonance frequency (0.82 THz) and two more frequencies far from the resonance (0.40 and 1.20 THz). (**d**) Schematic of the metamaterial absorber unit cell. A vector plot of electric current density on the surface of (**e**) the gold cross and (**f**) the VO_2_ film. (**g**) Distribution of the *x*-component of the normalized magnetic field (|H_x_|) in the spacer.

Metamaterial absorbers that can achieve unity absorptivity have attracted considerable attention since the first experimental demonstration^57^. Potential applications including, but are not limited to, IR camouflage, thermophotovoltaics, electromagnetic modulators and enhanced nonlinearity. At THz frequencies, in addition to the more conventional microelectromechanical systems (MEMS) based method^58, 59^, liquid crystal enabled tunable metamaterial absorber designs have been recently reported^17^. In Fig. 1(c), we illustrate a hybrid metamaterial absorber in which a SiO_2_ film serves as the spacer between an Au-cross array and the VO_2_ film that functions as a ground plane in the metallic phase. Figure 4(a) shows the σ_1_ dependence of the absorption spectra resulting from the metamaterial system, while the corresponding absorption properties of the VO_2_ and gold cross are illustrated in Fig. 4(b). These results reveal that, when the VO_2_ film is in the metallic phase, the optimized structure behaves like a perfect absorber with the achievement of unity absorptivity around 0.82 THz. More importantly, the absorption properties of the system closely depend on the conducting characteristics of the VO_2_ ground plane, which enables the creation of THz absorbers with dynamically tunable properties. To have a complete picture of the hybrid metamaterial absorber, in Fig. 4(c) we plot the corresponding power transmittance (*T*) and reflectance (*R*) at 0.82 THz as well as two more frequencies that are far from the resonance as a function of σ_1_. For the sake of comparison, *T* and *R* data for the bare VO_2_ film on a substrate are also provided. It can be seen that *T* (*R*) of the system at 0.82 THz is low (high) even when the VO_2_ is in its insulator phase, which can be attributed to the intrinsic electric resonance of the Au-cross. As the value of σ_1_ is increased, *R* at 0.82 THz dramatically decreases until both *T* and *R* reach near-zero values when σ_1_ → 1000 Ω^−1^ cm^−1^. For frequencies far away from the resonance (*e*.*g*., 0.40 and 1.20 THz), *T* decreases but *R* increases with increasing σ_1_, resulting from the fact that a rather weak interaction between the Au-cross and the VO_2_ film is expected. As these results suggest, all scattering parameters of the proposed metamaterial absorber can be purposely controlled, which is distinctly different from the reported tunable absorber devices^17, 59^ with fixed opaque ground plane and can therefore be highly beneficial for sophisticated manipulation of THz radiation. To confirm the perfect absorber mode, a vector plot of electric current density on the Au-cross and the VO_2_ film surface are shown in Fig. 4(e) and (f), respectively. The antiparallel induced currents on the two surfaces are evidence of a magnetic resonance, as is further illustrated in the magnetic field distribution in Fig. 4(g).

Given the fact that the response of natural materials to the magnetic component of an electromagnetic wave vanishes at THz frequencies and above, the ability of metamaterials to create artificial magnetism is of paramount importance. Indeed, the ability of metamaterials to support magnetic resonances has facilitated the achievement of various exotic phenomena, including negative refraction, cloak based invisibility, and the perfect absorber effect. Compared with SRRs, which are the primary metamaterial resonator utilized for achieving artificial magnetism at radio frequencies, the paired-strip design consisting of two conductive strips separated by a dielectric spacer has been employed to realize strong magnetic responses at optical frequencies^60^, because of its planar structure that is more amenable to micro- and nano-fabrication.

**Figure 5:**
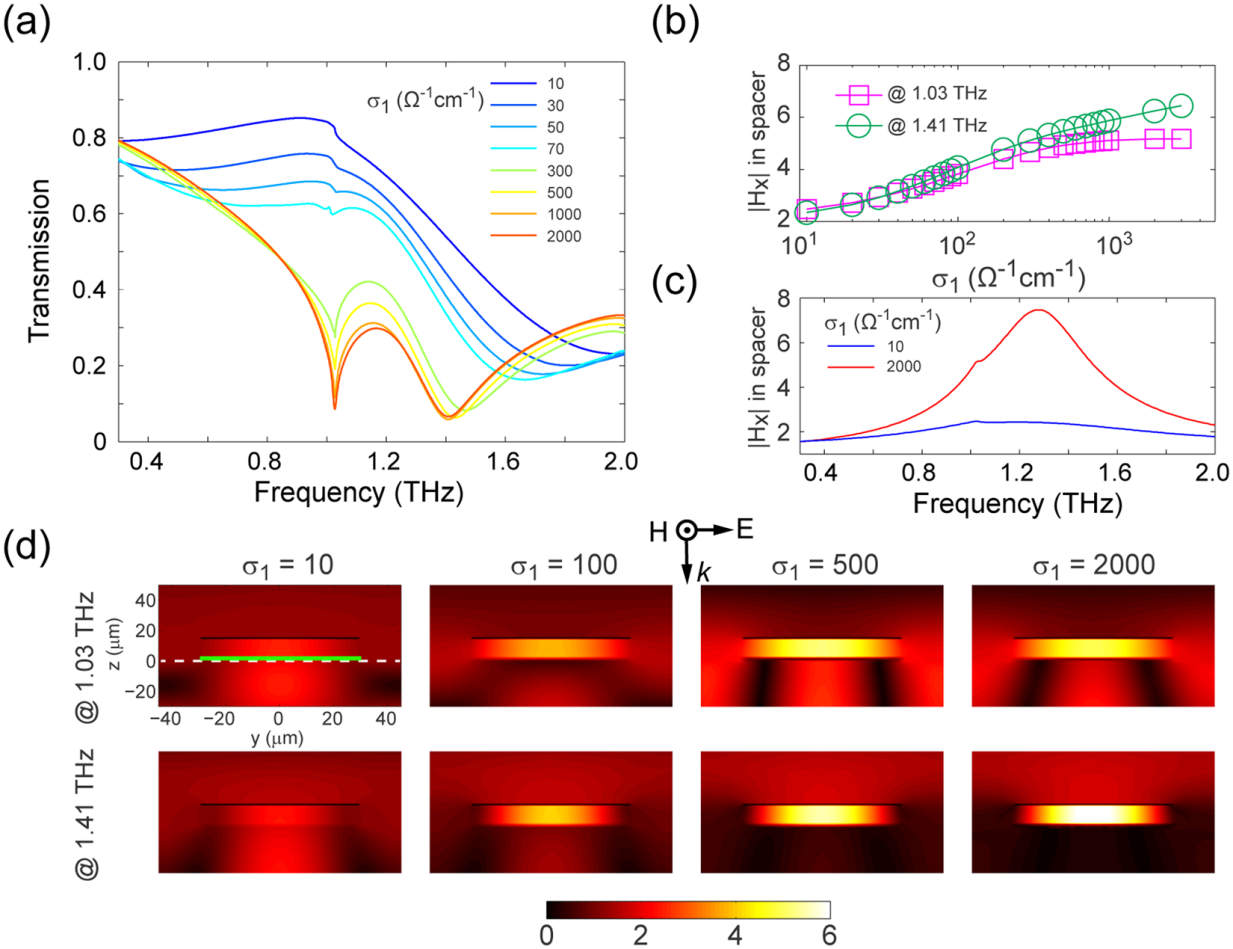
Au/VO_2_ paired-strip based active magnetic THz metamaterial. (**a**) Simulated transmission spectra. (**b**) The *x*-component of magnetic field (|H_x_|) at the geometric center of the spacer as a function of σ_1_. (**c**) The dispersion of (|H_x_|) when σ_1_ = 10 and 2000 Ω^−1^ cm^−1^. (**d**) The distribution of magnetic field at the two resonances (*i*.*e*., 1.03 and 1.41 THz) for a series of σ_1_ values. The white dashed line indicates the surface of the substrate and the green line represents the VO_2_ film. All field data was normalized to the magnetic field magnitude of the incident wave.

A hybrid metamaterial composed of a one-dimensional array of VO_2_/gold paired-strips is proposed (see Fig. 1(d)) to implement tunable magnetism at THz frequencies. First of all, the simulated transmission spectra shown in Fig. 5(a) indicate the largely controllable response of the system due to the IMT of VO_2_. In particular, when σ_1_ is greater than 300 Ω^−1^ cm^−1^, two transmission dips are identified around 1.03 and 1.41 THz, at where, however, the hybrid metamaterial with insulating VO_2_ (σ_1_ = 10 Ω^−1^ cm^−1^) is relatively transparent to the THz wave. In order to understand the underlying physical mechanism, the normalized magnetic field (|H_x_|) at the geometric center of the spacer is obtained at the resonances from a field probe and plotted as a function of σ_1_ in Fig. 5(b). Clearly, the magnetic field within the VO_2_/gold paired-strips is increasingly enhanced when there is a corresponding increase in σ_1_. In addition, as shown in Fig. 5(c), compared with the case with VO_2_ in the insulating state (σ_1_ = 10 Ω^−1^ cm^−1^), a broadband and pronounced enhancement of |H_x_| in the spacer is observed when σ_1_ = 2000 Ω^−1^ cm^−1^. Moreover, Fig. 5(d) shows the evolution of the magnetic field distribution in a cross-section plane orthogonal to the strips for different values of σ_1_. As σ_1_ is increased, a corresponding increase in the magnetic field confinement in the space between the VO_2_ and gold elements is observed. A comparison between the two figures in the most-right column of Fig. 5(d) unambiguously reveals the distinct mode of the two resonances arising from the permittivity difference between the substrate (sapphire) and superstrate (air).

## Discussion

Exhibiting lattice rearrangement or deformation at the atomic level, phase-transition materials have shown the capability of providing a large change in refractive index over a broad frequency range and consequently have been utilized to enable active photonic systems. Beyond the large conductivity variations that can be achieved during the IMT, as a classical transition metal oxide VO_2_ also exhibits hysteresis, a property associated with the first-order structural transition. Taking advantage of this hysteretic behavior, researches have demonstrated the memory process in VO_2_ integrated hybrid metamaterials^32, 43^. We emphasize that, though for simplicity no hysteretic characteristics have been taken into account in our designs, the proposed hybrid metamaterials are expected to manifest system history dependent THz responses, including the controllable memory effect. Besides temperature, other field regulation methods such as optical pumping, electric current and electric field induced phase transition of VO_2_ have been reported. In contrast to methods based upon thermal effects, optically-induced and field-effect-induced IMT can adjust VO_2_’s material parameters at a subpicosecond level. Consequently, the proposed hybrid metamaterials have the potential for ultrafast modulation of THz radiation.

Although it has been challenging to grow thick single phase VO_2_ due to the existence of a large number of states in the material, a variety of preparation procedures including pulsed laser deposition (PLD), molecular beam epitaxy (MBE), and sputtering have been utilized to achieve crystalline VO_2_ thin films. In particular, radio frequency (RF) sputtering, a relatively straightforward approach that requires controllable conditions (including an Ar and O_2_ mixture environment, substrate temperature and RF power) has been implemented to obtain high quality VO_2_ films^61–63^. Furthermore, we note that VO_2_ films with acceptable quality have been successfully grown on different substrates such as gold^43^ and glass^44^, which would offer more flexibility in the design of hybrid THz metamaterials. For instance, the metallic substrate may allow the metal/VO_2_/metal configuration to enable, for example, negative refraction^64^ and giant chirality^65^ at THz frequencies.

In summary, we have demonstrated that the integration of VO_2_ structures with conventional metallic resonating components can enable a class of highly tunable THz metamaterials. By presenting a series of proof-of-concept designs, we show that the insulator-to-metal transition of VO_2_ can facilitate dramatic response tuning of gap-loaded SRRs. More importantly, it was shown that the IMT of VO_2_ can also enable hybrid metamaterials exhibiting active Fano resonances, the perfect absorber effect, and artificial magnetism at THz frequencies. These responses can be dynamically tuned over a large scale, owing to the dramatic change in the THz electrical conductivity of VO_2_ enabled by its IMT. Considering the reported subpicosecond response times of VO_2_ under external stimulus, the proposed hybrid metamaterial systems may open up new avenues for highly tunable ultrafast THz devices.

## Methods

The permittivity of VO_2_ in the THz region can be described by the following Drude model: $\varepsilon (\omega )={\varepsilon }_{\infty }-\frac{{({\omega }_{{\rm{p}}}({\sigma }_{1}))}^{2}}{{\omega }^{2}+i\gamma \omega }$, where *ε*_∞_ is the permittivity at high frequency, *ω*_p_(*σ*_1_) is the conductivity dependent plasmon frequency and *γ* is the collision frequency^46^. On the other hand, both ${\omega }_{{\rm{p}}}^{2}$ and *σ*_1_ are proportional to the free carrier density. Therefore, the plasmon frequency at ${\sigma }_{1}^{^{\prime} }$ can be approximately expressed as ${\omega }_{{\rm{p}}}^{2}({\sigma }_{1}^{^{\prime} })=(\frac{{\sigma }_{1}^{^{\prime} }}{{\sigma }_{1}}){\omega }_{{\rm{p}}}^{2}({\sigma }_{1})$. By fitting the measured THz spectra shown in ref. 37, we determined that when *σ*_1_ = 3 × 10^3^ Ω^−1^ cm^−1^ (*ε*_∞_ = 12), the corresponding *ω*_p_ = 1.40 × 10^15^ rad/s, while *γ* = 5.75 × 10^13^ rad/s is assumed to be independent of *σ*_1_. These Drude model parameters agree well with the experimental results reported by other groups^46^. In addition, considering the roughly 1 μm thick skin depth of metallic-phase VO_2_ around 1.0 THz, 3-μm-thick VO_2_ structures were utilized to construct the hybrid resonators. Full-wave simulations were implemented using the commercial finite integration package CST Microwave Studio. For each type of hybrid metamaterial, a unit cell of the structure was simulated by employing periodic boundary conditions.

### Data availability

The data that support the findings of this study are available from the corresponding author on request.
